# Assessment of Biomechanical Parameters and Their Correlation During Barefoot Walking and Arch-Building Gait in Plantar Fasciitis Using a Dynamic Gait Analysis System: A Cross-Sectional Study

**DOI:** 10.7759/cureus.108016

**Published:** 2026-04-30

**Authors:** Banoth K Kumar, Shoibam Jenifa, Vineet T Abraham, Srikanth Muni, Purushotham Lingaiah, Timitrov P, Raunak Kumar

**Affiliations:** 1 Physical Medicine and Rehabilitation, All India Institute of Medical Sciences, Mangalagiri, Mangalagiri, IND; 2 Orthopaedics, All India Institute of Medical Sciences, Mangalagiri, Mangalagiri, IND; 3 Nephrology, All India Institute of Medical Sciences, Mangalagiri, Mangalagiri, IND

**Keywords:** arch-building gait, biomechanics, gait analysis, plantar fasciitis, plantar force

## Abstract

Background

Plantar fasciitis (PF) is one of the most common causes of heel pain and is associated with significant functional limitation and reduced quality of life. Alterations in foot biomechanics and plantar pressure distribution are considered key contributors. Short foot exercises effectively strengthen intrinsic foot muscles, improve arch support, and dynamic stability. We can incorporate the arch-building gait as a short-foot exercise. This study aims to evaluate and compare biomechanical parameters and plantar forces at the forefoot, midfoot, and heel among individuals with unilateral and bilateral PF and healthy individuals, using a dynamic gait analysis system. Additionally, it analyses the correlations during barefoot walking and arch-building gait.

Methods

This cross-sectional study was conducted at the Department of Physical Medicine and Rehabilitation (PMR), All India Institute of Medical Sciences (AIIMS), Mangalagiri, India, over 15 months. A total of 141 participants were included: 47 with unilateral PF, 47 with bilateral PF, and 47 healthy individuals. Gait analysis was performed using the Diers Formetric 4D system (DIERS, Biomedical Solutions, Germany) to assess biomechanical gait parameters, as well as plantar force distribution at the forefoot, midfoot, and heel during barefoot walking and arch-building gait. Statistical analysis was carried out using ANOVA, the chi-square test, and Pearson's correlation coefficient, with p-values < 0.05 considered statistically significant.

Results

A total of 141 participants were included, with 47 in each of the following groups: unilateral (U/L), bilateral (B/L), and normal. No significant differences in biomechanical gait parameters were observed among the groups during barefoot walking. In contrast, during arch-building gait, significant differences were present in cadence and antero-posterior (A/P) centre of pressure. For barefoot walking, a significant difference in midfoot plantar force was found between the unilateral and bilateral PF groups, whereas no significant differences emerged during arch-building gait. Strong-to-moderate positive correlations were observed between barefoot walking and arch-building gait parameters across all groups.

Conclusion

Individuals with unilateral and bilateral PF show near-normal gait during barefoot walking. Arch-building gait reveals subtle biomechanical changes and improves plantar load distribution. These findings support that the arch-building gait activates the intrinsic foot muscles and can serve as both a sensitive assessment tool and a potential rehabilitation strategy.

## Introduction

Plantar fasciitis (PF) is the most common cause of heel pain in outpatient clinics, accounting for a large proportion of foot-related healthcare visits [1]. It significantly impairs mobility and quality of life. The condition involves inflammation and degeneration of the plantar fascia, a thick connective tissue band that originates from the medial calcaneal tuberosity and runs along the bottom of the foot. This tissue is essential for supporting the medial arch and enabling proper foot function during standing and walking.

Clinically, PF presents as pain and stiffness in the heel and medial arch. The main symptom is a sharp pain during the first steps in the morning or after prolonged rest [2]. This "first-step pain" is caused by the plantar fascia tightening during inactivity. Symptoms may briefly improve after a few steps, but pain usually increases with extended weight-bearing activity as the day progresses [3]. Symptom severity and duration vary and can disrupt work, leisure, and daily routines.

PF is diagnosed mainly on clinical assessment, including patient history and examination. Patients usually describe gradually increasing heel pain at the base of the heel, often without a specific injury. Tenderness is reproduced by pressing on the medial calcaneal tubercle and along the plantar fascia. Imaging methods, such as ultrasound or MRI, are reserved for unusual or persistent cases and are not routinely necessary [4,5].

Most patients respond well to conservative treatments. These include stretching exercises for the plantar fascia and Achilles tendon, cryotherapy, thermotherapy, electrical therapies, nonsteroidal anti-inflammatory drugs (NSAIDs), extracorporeal shock wave therapy (ESWT), and foot orthoses such as insoles [3-6]. These treatments aim to decrease inflammation, ease pain, increase flexibility, and restore function.

Interventions targeting the strengthening of the foot's intrinsic muscles, such as the short-foot exercise (SFE), and the arch-building gait, increase intrinsic plantar muscle activation. It also enhances arch support and contributes to stability during walking. Coupled with the improvements in proprioception and balance, these exercises can potentially decrease stress on the plantar fascia and reduce pain severity [7].

More research is needed on the effectiveness of short-foot exercises for reducing pain, improving dynamic stability, and enhancing performance in people with PF.

Thus, this study aims to evaluate and compare biomechanical parameters and plantar forces at the forefoot, midfoot, and heel among individuals with unilateral and bilateral PF, as well as healthy individuals, using a dynamic gait analysis system during barefoot walking and arch-building gait.

## Materials and methods

Study design and setting

This cross-sectional study was conducted in the outpatient department (OPD) of the Department of Physical Medicine and Rehabilitation (PMR) at AIIMS Mangalagiri, India, over 15 months, from 24-11-2024 to 24-02-2026.

Eligibility criteria

During the study, all individuals aged 18 or older visiting the OPD of the Department of PMR were screened for eligibility. Patients with PF, based on history and clinical exam, and with heel pain lasting more than four months, were included. Subjects were excluded for traumatic events, any prior surgery or fracture of the lower limb, limb length discrepancy of one centimetre (cm) or more, BMI above 35 kg/m^2^, rheumatological conditions, disorders of the nervous system, calcaneal spur, or any intralesional injections. Participants were divided into three groups: unilateral PF, bilateral PF, and a healthy group without heel pain.

Sample size

Based on Yoo et al. [8], we considered a minimum mean difference of 7.6 N/cm² in heel maximum pressure among groups, with a pooled standard deviation of 10 N/cm². We aimed for 90% power and a 5% type I error rate with Bonferroni correction. The sample size was estimated using a comparison of two independent samples, with the alpha level adjusted for multiple comparisons. Each group needed 47 subjects, for a total sample size of 141.

Method

All the subjects who met the inclusion and exclusion criteria were enrolled in the study. Informed written consent was taken from all enrolled subjects. Subjects complaining of heel pain for more than four months and diagnosed with PF clinically were taken as PF cases, while normal subjects with no heel pain were taken as the normal group. The Diers Formetric 4D (DIERS, Biomedical Solutions, Germany) gait analysis system was used to measure biomechanical parameters, including kinetic (maximum force) and kinematic parameters, such as gait cycle, step parameters, and centre of pressure (COP) in terms of gait line length, antero-posterior (A/P) position, and lateral position. The system consists of a foot platform integrated into a treadmill. All participants walked barefoot. They were asked to walk on the treadmill at a relaxed posture at 1.8 km/h for five minutes. Participants were educated on the short-foot exercise before the arch-building motion was introduced. Participants were asked to walk with the arch of their feet raised by pulling the big toe toward the heel, which shortens the foot for about 10 minutes. An arch-building gait was achieved by flexing the metatarsophalangeal and proximal interphalangeal joints during gait while minimising distal interphalangeal joint flexion. Reassessment was done 10 minutes after training on the arch-building gait. After practising this gait, participants were asked to walk on the treadmill at 1.8 km/h for five minutes. Each subject's gait analysis lasted 20 minutes. The principal investigator recorded and interpreted a computer-generated report of biomechanical parameters for all participants during barefoot walking and arch-building gait and then compared them. Multiple biomechanical parameters, including gait parameters and maximum force at forefoot, midfoot, and heel, were measured in both the PF cases and normal groups during barefoot and arch-building gait.

Outcome measures

The primary goal was to evaluate biomechanical parameters, with a focus on step characteristics, such as foot rotation at (°), step length in centimetres (cm), step time in milliseconds (ms), and cadence in steps per minute (steps/min). Gait cycle metrics included stance phase (%), load response (%), single support (%), pre-swing (%), and swing phase (%). Additionally, the COP was recorded in terms of gait line length and A/P and lateral positions, in centimetres (cm). Maximum force measurements at the forefoot, midfoot, and heel were also obtained in Newton (N).

Statistical analysis

Categorical variables were presented as numbers and percentages (%), while quantitative data were shown as means ± SD. To analyse the results, various statistical tests were employed. ANOVA with Tukey's honest significance difference (Tukey's HSD) post-hoc test for quantitative variables, and the chi-square test for qualitative variables. Additionally, Pearson's correlation coefficient (r) was used to assess the relationship between biomechanical parameters during barefoot walking and arch-building gait. Data entry was performed in Microsoft Excel (Microsoft® Corp., Redmond, WA), and the final analysis was conducted using the Statistical Product and Service Solutions (SPSS, version 25.0; IBM SPSS Statistics for Windows, Armonk, NY). For statistical significance, a p-value of less than 0.05 was considered statistically significant.

## Results

A total of 141 participants were included in the study, with 47 participants each in the unilateral, bilateral, and normal groups. There was a statistically significant difference in age among the three groups (ANOVA, p = 0.003). After post-hoc analysis and adjustment for multiple comparisons with a Bonferroni-corrected significance level of 0.017, the unilateral group (42.74 ± 10.72 years) was significantly older than the bilateral group (37.32 ± 5.91 years; p = 0.004). Gender distribution differed significantly among the groups (p = 0.01). The proportion of females was higher in the unilateral group (76.60%) than in the normal group (48.94%; p = 0.006*). There were no statistically significant differences in weight and height among the groups. Overall, the groups were comparable in terms of weight and height, whereas significant differences were observed in age and gender distribution (Table 1).

**Table 1:** Comparison of baseline characteristics between unilateral (U/L) PF, bilateral (B/L) PF, and normal subjects. † ANOVA followed by post-hoc test (Tukey HSD test) with Bonferroni-corrected significance level of 0.017; *Chi-square test followed by post-hoc test (pairwise Z-test) with Bonferroni-corrected significance level of 0.017; U/L = unilateral; B/L = bilateral; PF = plantar fasciitis; A/P = antero-posterior

Baseline characteristics	Unilateral (n = 47)	Bilateral (n = 47)	Normal (n = 47)	P value
Age (years)	42.74 ± 10.72	37.32 ± 5.91	38.23 ± 6.65	0.003 †
				U/L vs B/L: 0.004; U/L vs Normal: 0.020; B/L vs Normal: 0.846
Gender				0.01 *
Female	36 (76.60%)	34 (72.34%)	23 (48.94%)	U/L vs B/L: 0.636*; U/L vs Normal: 0.006*; B/L vs Normal: 0.02*
Male	11 (23.40%)	13 (27.66%)	24 (51.06%)	
Weight (kg)	71.43 ± 11.02	69.29 ± 11.88	71.28 ± 12.1	0.615 †
Height (cm)	159.21 ± 9.18	163 ± 7.79	161.89 ± 9.06	0.099 †

Barefoot walking parameters were similar across the unilateral, bilateral, and normal groups, with no statistically significant differences in any measured gait parameters (Table 2).

**Table 2:** Comparison of biomechanical parameters during barefoot walking in unilateral (U/L) PF, bilateral (B/L) PF, and normal subjects. † ANOVA; U/L = unilateral; B/L = bilateral; PF = plantar fasciitis; A/P = antero-posterior

Gait parameters in barefoot walking	Unilateral (n = 47)	Bilateral (n = 47)	Normal (n = 47)	P value
Foot rotation (⁰)	16.98 ± 6.34	17.94 ± 4.58	18.69 ± 4.46	0.281 †
Step length (cm)	32.17 ± 6.06	30.96 ± 3.64	31.48 ± 4.44	0.475 †
Step time (ms)	553.83 ± 72.39	567.7 ± 51.4	579.61 ± 77.56	0.189 †
Cadence (steps/min)	109.34 ± 14.5	105.64 ± 9.77	106.66 ± 14.22	0.365 †
Stance phase (%)	68.74 ± 3.1	68.85 ± 2.67	69.36 ± 1.91	0.473 †
Load response (%)	19.32 ± 2.97	19.05 ± 1.67	19.03 ± 1.92	0.79 †
Single support (%)	30.81 ± 2.47	30.89 ± 1.4	30.94 ± 1.77	0.948 †
Pre-swing (%)	19 ± 2.34	19.32 ± 1.77	19.77 ± 2.3	0.227 †
Swing phase (%)	30.57 ± 2.3	30.68 ± 2.42	30.67 ± 1.88	0.968 †
Gait line length (cm)	8.83 ± 2.17	8.86 ± 1.63	9.45 ± 2.26	0.253 †
A/P position (cm)	2.59 ± 4.48	1.58 ± 0.81	2.24 ± 3.68	0.345 †
Lateral position (cm)	4.41 ± 3.58	4.35 ± 5.54	4.45 ± 3.49	0.994 †

During arch-building gait, evaluation revealed a statistically significant difference in cadence among the three groups (p = 0.034), after post-hoc analysis with Bonferroni correction (significance level: 0.017); no significant differences were observed between the groups. A statistically significant difference was also found in A/P position (p = 0.002). Post-hoc analysis showed a significant difference between the bilateral and normal groups (p = 0.001), with the bilateral group showing higher A/P values (4.2 ± 4.39 cm) compared to the normal group (1.77 ± 1.64 cm). No significant differences were observed between unilateral and bilateral groups or between unilateral and normal groups. There were no statistically significant differences among the three groups across other parameters (Table 3).

**Table 3:** Comparison of biomechanical parameters during arch-building gait in unilateral (U/L) PF, bilateral (B/L) PF, and normal subjects. † ANOVA followed by post-hoc test (Tukey's honest significance difference (HSD) test) with Bonferroni-corrected significance level of 0.017; U/L = unilateral; B/L = bilateral; PF = plantar fasciitis; A/P = antero-posterior

Arch-building gait	Unilateral (n = 47)	Bilateral (n = 47)	Normal (n = 47)	P value
Foot rotation (⁰)	14.64 ± 7.19	14.82 ± 5.69	15.01 ± 5.33	0.957†
Step length (cm)	28.7 ± 6.16	29.69 ± 3.91	30.31 ± 4.1	0.27†
Step time (ms)	525.77 ± 73.71	539.31 ± 44.2	551.31 ± 84.63	0.209†
Cadence (steps/min)	119.04 ± 22.4	113.77 ± 7.77	110.43 ± 14.25	0.034†
				U/L vs B/L: 0.248
				UL vs Normal: 0.027
				B/L vs Normal: 0.569
Stance phase (%)	67.68 ± 2.88	69.12 ± 3.73	68.72 ± 2.51	0.069†
Load response (%)	18.68 ± 2.36	18.73 ± 2.95	18.5 ± 2.28	0.897†
Pre-swing (%)	18.96 ± 2.15	19.39 ± 2.96	18.9 ± 2.33	0.583†
Swing phase (%)	31.7 ± 2.48	30.57 ± 3.14	31.27 ± 2.51	0.133†
Gait line length (cm)	7.57 ± 1.92	8.05 ± 2.43	8.08 ± 2.11	0.446†
A/P position (cm)	3.28 ± 3.02	4.2 ± 4.39	1.77 ± 1.64	0.002†
				U/L vs B/L: 0.358
				UL vs Normal: 0.062
				B/L vs Normal: 0.001
Lateral position (cm)	13.45 ± 17.47	11.87 ± 20.24	6.5 ± 6.36	0.088†

During barefoot walking, the maximum forces at the forefoot and heel did not differ significantly among the three groups. However, a statistically significant difference was observed in the midfoot (p = 0.028). After post-hoc analysis with Bonferroni correction (significance level: 0.017), no significant differences were found among the three groups. In contrast, during the arch-building gait, no statistically significant differences in maximum force were observed among the groups for the forefoot (p = 0.264), midfoot (p = 0.411), or heel (p = 0.144). Additionally, all pairwise comparisons within these regions were non-significant (Table 4).

**Table 4:** Comparison of the maximum force during barefoot walking and arch-building gait in unilateral (U/L) PF, bilateral (B/L) PF, and normal subjects. † ANOVA followed by post-hoc test (Tukey's honest significance difference (HSD) test) with Bonferroni-corrected significance level of 0.017; U/L = unilateral; B/L = bilateral; PF = plantar fasciitis

Force (N)	Unilateral (n = 47)	Bilateral (n = 47)	Normal (n = 47)	P value
Barefoot walking				
Forefoot	147.87 ± 51.21	158.56 ± 52.49	151.93 ± 50.11	0.595 †
Midfoot	102.96 ± 51.57	76.39 ± 37.86	87.18 ± 52.56	0.028 †
				U/L vs B/L: 0.022
				UL vs Normal: 0.249
				B/L vs Normal: 0.519
Heel	191.89 ± 63.75	201.29 ± 39.88	180.4 ± 49.99	0.155 †
Arch-building gait				
Forefoot	116.85 ± 61.98	106.63 ± 38.45	123.5 ± 47.4	0.264 †
Midfoot	151.64 ± 88.22	131.03 ± 58.19	142.19 ± 74.9	0.411 †
Heel	210.28 ± 73.1	215 ± 55.59	191.73 ± 49.08	0.144 †

When evaluating the correlations between biomechanical parameters of barefoot walking and arch-building gait across the three groups, there were positive, strong-to-moderate, and statistically significant associations for most parameters. However, no statistically significant correlation was noted in the stance phase (r = 0.168, p = 0.259) and A/P position (r = 0.274, p = 0.062) in the unilateral PF group. In the bilateral PF group, no statistically significant correlation was observed for stance phase (r = 0.190, p = 0.200), A/P position (r = -0.052, p = 0.726), lateral position (r = -0.027, p = 0.855), and forefoot force (r = 0.241, p = 0.102). Additionally, for the normal group, no statistically significant correlations were observed for A/P position (r = 0.169, p = 0.258) or lateral position (r = 0.272, p = 0.064) (Table 5).

**Table 5:** Correlation of biomechanical parameters between barefoot walking and arch-building gait in unilateral (U/L), bilateral (B/L) PF, and normal subjects. Pearson's correlation coefficient; U/L = unilateral; B/L = bilateral; PF = plantar fasciitis; A/P = antero-posterior

Biomechanical parameters	Unilateral		Bilateral		Normal	
	r value	P value	r value	P value	r value	P value
Foot rotation (°)	0.722	<0.0001	0.62	<0.0001	0.535	0.0001
Step length (cm)	0.654	<0.0001	0.467	0.001	0.688	<0.0001
Step time (ms)	0.66	<0.0001	0.468	0.001	0.731	<0.0001
Cadence (steps/min)	0.612	<0.0001	0.395	0.006	0.742	<0.0001
Stance phase (%)	0.168	0.259	0.19	0.200	0.471	0.001
Load response (%)	0.409	0.004	0.662	<0.0001	0.519	0.0002
Single support (%)	0.463	0.001	0.653	<0.0001	0.625	<0.0001
Pre-swing (%)	0.679	<0.0001	0.6	<0.0001	0.524	0.0002
Swing phase (%)	0.297	0.043	0.476	0.0007	0.443	0.002
Gait line length (cm)	0.625	<0.0001	0.583	<0.0001	0.783	<0.0001
A/P position (cm)	0.274	0.062	-0.052	0.726	0.169	0.258
Lateral position (cm)	0.293	0.046	-0.027	0.855	0.272	0.064
Force at the forefoot	0.401	0.005	0.241	0.102	0.477	0.001
Force at the midfoot	0.469	0.001	0.446	0.002	0.75	<0.0001
Force at the heel	0.595	<0.0001	0.506	0.0003	0.684	<0.0001

## Discussion

The present study examined the differences in biomechanical parameters, focusing on gait characteristics and plantar force distribution at the forefoot, midfoot, and heel among individuals with unilateral and bilateral PF and healthy individuals during barefoot walking and arch-building gait.

Arch-building gait is a short-foot exercise that involves pulling the first metatarsal head toward the heel, thereby increasing the medial longitudinal arch and reducing strain on it [9].

A total of 141 participants were included in the study, with 47 participants in each of the unilateral, bilateral, and normal groups. A statistically significant age difference was observed among the groups, with the unilateral PF group being older than the bilateral PF group. This supports the association between increasing age and PF, likely due to degenerative changes in the plantar fascia, such as reduced elasticity and increased stiffness [10,11]. Recent studies continue to support age as a contributing factor, emphasising cumulative mechanical stress and tissue degeneration over time [12].

Gender distribution also differed significantly, with a higher proportion of females in both PF groups compared to the normal group. This is consistent with both classical and recent findings indicating a higher prevalence of PF among females, potentially due to biomechanical and lifestyle-related factors [12,13].

No significant differences in height or weight were found between groups, indicating minimal confounding by anthropometric variables. In this study, no statistically significant differences were identified among the three groups for any gait parameters during barefoot walking. The parameters remained comparable across groups. This evidence suggests that individuals with PF may maintain near-normal gait patterns during habitual walking, possibly through compensatory mechanisms that minimise pain or mechanical stress. Previous studies have similarly reported that PF does not necessarily alter global spatiotemporal parameters but instead affects more subtle aspects of foot function and pressure patterns [14,15].

However, the arch-building gait showed significant differences in selected parameters. A significant difference in cadence was observed across the three groups. Although no significant differences were observed among the groups, the unilateral group's cadence was higher than that of both the bilateral and normal groups. This may represent a compensatory strategy intended to reduce stance duration and, consequently, plantar fascia loading. Additionally, a significant difference in A/P position was observed, particularly between the bilateral PF and normal groups. In partial support of the findings of a previous study by Yoo et al. [8], where there was decreased gait line length, single support line, and A/P position of COP, the similar finding in this study could be due to redistribution of the COP during the arch-building gait.

Regarding the maximum plantar force distribution during barefoot walking, a significant difference in midfoot force was observed among the three groups. Although no statistically significant difference was found in the pairwise comparison, the midfoot force in the unilateral group was higher than in both the bilateral and normal groups. This suggests altered plantar pressure and force distribution, as well as compensatory mechanisms. Recent evidence indicates that PF is linked to abnormal plantar pressure and force patterns. One study observed increased pressure in the midfoot and a compensatory redistribution away from painful areas [8]. Additionally, Balaji et al. [16] reported a shift in pressure from the hindfoot to the forefoot, resulting in anterior load transfer. A previous study also reported that only 14% of the pressure was applied to the rear foot in patients with PF [17]. These changes are considered to contribute to symptom persistence and altered biomechanics.

In contrast to the above, no significant differences in plantar force at any of the three regions were observed during arch-building gait in this study, indicating that active arch engagement may normalise plantar force distribution. This supports recent rehabilitation approaches that focus on intrinsic foot-muscle strengthening and arch control [18,19]. Recent studies highlight the importance of dynamic foot control and intrinsic muscle activation in maintaining arch integrity, with deficits associated with altered gait mechanics in PF. Therefore, arch-building tasks may provide a more sensitive functional assessment and serve as a rehabilitative strategy, such as short foot exercises, for managing plantar fascia-related dysfunction.

This study also aimed to identify the correlations between biomechanical parameters and plantar force distribution across groups during barefoot and arch-building gait. It revealed strong positive associations between barefoot walking and arch-building gait in several biomechanical measures across all groups.

In cases of unilateral PF, strong correlations were observed with parameters such as foot rotation, step length, and cadence, indicating consistent gait patterns and suggesting that gait adaptability remains intact despite localised pathology. Similarly, bilateral PF showed strong correlations in load response, foot rotation, and single-support phases, implying preserved temporal organisation of gait despite the condition. In the normal group, even stronger correlations were observed across most parameters, reflecting steady, efficient gait mechanics.

However, the absence of a significant correlation between A/P and lateral position parameters across all three groups during barefoot walking and arch-building gait suggests that these variables may be more sensitive to condition-specific or pathological influences, emphasising their potential role in detecting gait alterations.

Strengths and limitations

Our study utilises objective dynamic gait analysis with an advanced system to ensure accurate and consistent measurement of biomechanical parameters. Including both functional (barefoot walking) and rehabilitative (arch-building gait) conditions allows for a more thorough assessment of gait adaptations.

The cross-sectional design of the study limits causal conclusions. The groups are not age- or gender-matched, which hampers more accurate comparisons. Pain severity, duration, and activity levels were not examined, though they may affect gait adaptations. Furthermore, the lack of plantar pressure distribution across different regions of the plantar surface limits a deeper understanding of biomechanics.

## Conclusions

This study shows that individuals with unilateral and bilateral PF maintain near-normal biomechanical parameters during barefoot walking, while subtle parameter alterations and near-normal plantar force distribution become evident during arch-building gait. The findings highlight the role of intrinsic foot muscle activation in normalising plantar load distribution and suggest that arch-building gait may serve as both a sensitive assessment tool and an efficient rehabilitation strategy for PF. Additionally, positive correlations between barefoot walking and arch-building gait across most biomechanical parameters indicate preserved gait patterns in PF, while A/P and lateral position variables may serve as sensitive indicators of subtle gait alterations.
